# Transmembrane lectins in cancer immunity: emerging drivers of myeloid-mediated immunosuppression

**DOI:** 10.3389/fimmu.2026.1952520

**Published:** 2026-09-08

**Authors:** Gonçalo Trindade, Angelina Sá Palma, Catarina Brito

**Affiliations:** 1 iBET, Instituto de Biologia Experimental e Tecnológica Oeiras, Oeiras, Portugal; 2 Instituto de Tecnologia Química e Biológica António Xavier, Universidade NOVA de Lisboa, Oeiras, Portugal; 3 UCIBIO – Applied Molecular Biosciences Unit, Departamento de Química, Faculdade de Ciências e Tecnologia, Universidade NOVA de Lisboa, Caparica, Portugal; 4 Associate Laboratory i4HB - Institute for Health and Bioeconomy, Faculdade de Ciências e Tecnologia, Universidade NOVA de Lisboa, Caparica, Portugal

**Keywords:** cancer immunotherapy, C-type lectin receptor (CLR), myeloid immunosuppression, Siglecs, transmembrane lectins, tumor glycosylation, tumor microenvironment (TME), tumor-associated macrophage (TAM)

## Abstract

Immune evasion in cancer is sustained by coordinated interactions between malignant, stromal, and immune cells within the tumor microenvironment. Among these, myeloid populations are major regulators of immunosuppression, but the diverse signals that shape their functional states remain insufficiently integrated within current conceptual frameworks. Altered glycosylation is a recurrent feature of cancer that reshapes the ligands available to lectin receptors. Within this landscape, transmembrane lectins expressed by myeloid cells occupy a distinctive mechanistic position, coupling extracellular glycan recognition to intracellular signaling programs that shape myeloid-cell function. In this review, we integrate evidence from tumor glycobiology, myeloid immunology, receptor signaling, experimental models, and therapeutic development to examine how C-type lectin receptors and Siglecs contribute to myeloid-mediated immune suppression. We discuss how these receptors influence macrophage and dendritic-cell states, antigen presentation, phagocytosis, cytokine production, metabolism, and immune-checkpoint regulation, while emphasizing the dependence of these outcomes on ligand context, receptor architecture, cellular identity, and tissue organization. We further consider how receptor redundancy, overlapping glycan specificities, and model-dependent effects complicate the interpretation and therapeutic targeting of lectin pathways. Finally, we discuss emerging approaches directed at lectin–glycan interactions and downstream signaling, together with their potential to complement established immunotherapies. By bringing together concepts that are often considered separately, this review positions transmembrane lectins as important integrators of the tumor glycan landscape and myeloid immune regulation, while defining key questions that must be addressed to translate glyco-immune mechanisms into effective cancer therapies.

## 1 Introduction

Tumor-associated glycosylation has emerged as an important regulator of cancer immunity, yet how glycan alterations are sensed by myeloid cells and translated into immunosuppressive states remains fragmented across glycobiology and tumor immunology (1–3). Transmembrane lectins occupy a central position at this interface by coupling extracellular glycan recognition to intracellular signaling (2, 4). This review focuses on myeloid-expressed transmembrane lectins, particularly C-type lectin receptors and Siglecs, while considering soluble lectins and broader glycosylation mechanisms where relevant to the biological and mechanistic context.

## 2 The tumor microenvironment in immune dysfunction

The ability to evade immune destruction is a defining hallmark of cancer, reflecting how tumor progression is shaped not only by tumor-intrinsic alterations but also by the active subversion of host immunity (5, 6). During tumorigenesis, immune surveillance can progressively give way to immune tolerance and escape through the elimination, equilibrium, and escape phases of cancer immunoediting (7–9).

Importantly, immune failure often reflects active immunosuppression rather than an absence of immune activity: tumors can remain immune-infiltrated while immune cells are functionally reprogrammed toward regulatory or tolerogenic states that dampen effector responses and support tumor growth (10–12). These programs are reinforced by local cytokines, metabolic constraints, and cell–cell interactions within the tumor microenvironment (TME) (12–14). Thus, immune dysfunction in cancer reflects an actively maintained state, arising from the integration of tumor-derived and microenvironmental cues that limit effective anti-tumor immunity (15, 16).

Immunosuppressive states within the TME exhibit substantial heterogeneity across tumors, reflecting differences in both immune composition, functional state and spatial organization that influence disease progression and therapeutic response (12, 17, 18).

Tumors are often broadly classified as “immune-inflamed” (“hot”) or “immune-excluded” (“cold”), based on the presence and spatial distribution of tumor-infiltrating T lymphocytes, with immune-inflamed tumors generally displaying greater cytotoxic T lymphocyte (CTL) infiltration and immune-excluded tumors showing limited T-cell infiltration (12, 18). However, this classification only partially captures immune function, as CTL infiltration does not necessarily reflect effective anti-tumor immunity. Instead, activation state, differentiation trajectories, and suppressive phenotypes are also critical determinants of outcome (17, 19–21).

**Figure 1:**
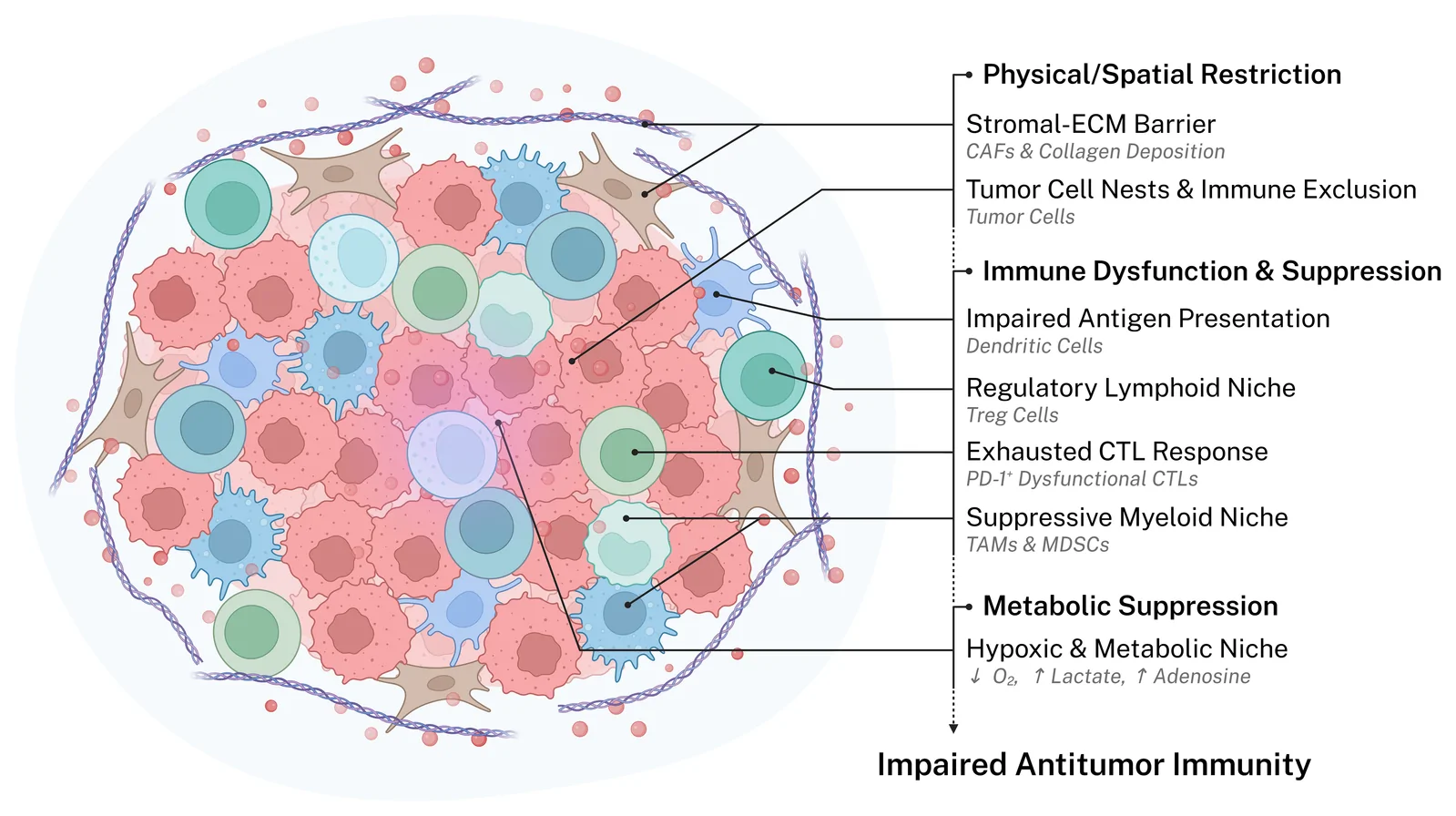
Mechanisms of immune dysfunction in the tumor microenvironment. Schematic representation of a heterogeneous tumor microenvironment containing tumor cell nests, cancer-associated fibroblasts (CAFs), collagen-rich extracellular matrix (ECM), dendritic cells, cytotoxic T lymphocytes (CTLs, CD8^+^ T cells), regulatory T cells (Tregs), tumor-associated macrophages (TAMs) and myeloid-derived suppressor cells (MDSCs). Annotated regions highlight key dysfunctional niches, including stromal–ECM immune exclusion, impaired antigen presentation, regulatory lymphoid accumulation, exhausted CTL responses, suppressive myeloid infiltration and a hypoxic metabolic niche characterized by reduced O_2_, lactate accumulation and adenosine enrichment. These mechanisms are organized into physical/spatial restriction, immune dysfunction and suppression, and metabolic suppression, illustrating their convergence on impaired antitumor immunity (15, 25, 54).

Multiple mechanisms contribute to this heterogeneity and to the impairment of immune function within the TME (**Figure 1**) (15, 22). These include defective antigen presentation, sustained inhibitory checkpoint signaling (e.g., PD-1/PD-L1), and metabolic constraints such as hypoxia, nutrient depletion, and accumulation of immunosuppressive metabolites such as lactate and adenosine (12, 14, 15). In addition, stromal cells such as cancer-associated fibroblasts (CAFs) and the extracellular matrix (ECM) further regulate immune recruitment, localization, and function through both biochemical and physical cues (23–26).

Rather than acting independently, these signals are integrated within spatially organized niches through cell localization, cell-cell interactions, and coordinated receptor-ligand engagement (12, 15, 24, 25). This organization shapes the tumor “immune contexture”, encompassing the type, density, localization and functional orientation of immune infiltrates, and thereby constrains immune accessibility and responsiveness (17, 27, 28).

Consequently, TME heterogeneity contributes to variability in prognosis and therapeutic response, while exposing distinct regulatory axes that may be therapeutically exploited (17, 18, 29).

Historically, cancer therapies have primarily targeted tumor-intrinsic pathways (5, 6). In breast cancer, this includes estrogen/progesterone receptor- and human epidermal growth factor receptor 2 (HER2)-directed strategies, which provide substantial clinical benefit but do not eliminate relapse or microenvironment-mediated adaptation (30–33).

Recognition of immune control of tumor progression drove the development of immunotherapy, including PD-1/PD-L1 checkpoint blockade, although responses remain heterogeneous and restricted to selected patients and tumor contexts (19, 20, 34–37).

Nevertheless, current immunotherapeutic strategies remain largely T cell–centric, whereas myeloid populations can actively sustain immunosuppressive states through regulation of antigen presentation, cytokine production, and tissue remodeling (12, 20, 38–41). These limitations highlight the need to identify upstream regulatory mechanisms that coordinate immune suppression within the TME (12, 16, 38).

## 3 Myeloid compartment in cancer immunosuppression

### 3.1 Myeloid populations in the tumor microenvironment

The TME is densely infiltrated by heterogeneous myeloid populations that integrate malignant, stromal, and metabolic cues and critically condition adaptive immune responses (11, 12, 42, 43). Although antitumor immunity is often framed through a lymphocyte-centric perspective, myeloid cells can determine whether T-cell responses are effectively initiated or functionally suppressed, positioning them as central regulators of immune tone and disease progression (38, 42, 43).

Circulating monocytes are a major source of tumor-infiltrating myeloid cells (44, 45). Upon recruitment via chemokine axes such as CCL2-CCR2, they can differentiate into tumor-associated macrophages (TAMs), monocyte-derived dendritic cells (moDCs), or monocytic myeloid-derived suppressor cells (M-MDSCs) (42, 44). In established tumors, sustained exposure to factors such as colony stimulating factor 1 (CSF1) and interleukin 6 (IL-6), together with stromal and metabolic cues, can skew differentiation toward immunoregulatory states (12, 43, 44).

Macrophages, often the dominant myeloid population within tumors, function as central coordinators of tissue remodeling and immune suppression (38, 45). The TAM compartment includes both monocyte-derived and tissue-resident populations, contributing to heterogeneous ontogenies and functional states (44, 46, 47). High TAM density and TAM-associated transcriptional programs have been linked to adverse outcomes across solid tumors, including breast cancer (38, 44, 45).

TAMs further exhibit spatial specialization across hypoxic, invasive, and perivascular niches linking their functional state to local microenvironment context (13, 45).

Dendritic cells (DCs), traditionally defined by their antigen-presenting capacity, provide another critical interface between innate and adaptive immunity (12, 48). Conventional type 1 DCs (cDC1), required for cross-priming of CD8+ T cells, are often excluded or functionally impaired in tumors, while other DC subsets acquire tolerogenic features that limit T-cell priming (11, 48, 49).

Myeloid-derived suppressor cells (MDSCs), including monocytic (M-MDSC) and polymorphonuclear (PMN-MDSC) subsets, expand under chronic inflammatory conditions and suppress T-cell responses through mechanisms including arginase-1 activity, nitric oxide production, and reactive oxygen species (39, 42).

Across these populations, functional plasticity enables continual adaptation to oxygen, metabolites, cytokines, and stromal cues (13, 43). Reciprocal crosstalk with tumor, fibroblast, endothelial, and ECM compartments further reinforces these states, creating self-sustaining circuits of immune suppression and tumor progression (13, 23, 43, 45). This plasticity and networked organization increase the robustness of tumor-associated immunosuppression that undermines therapeutic targeting (38, 43).

Within this compartment, macrophages are uniquely positioned due to their abundance, longevity, spatial organization, and capacity to integrate diverse environmental signals, motivating closer examination of TAM immunosuppressive function (38, 43, 45).

### 3.2 Tumor-associated macrophages: plasticity and immunosuppressive function

**Figure 2:**
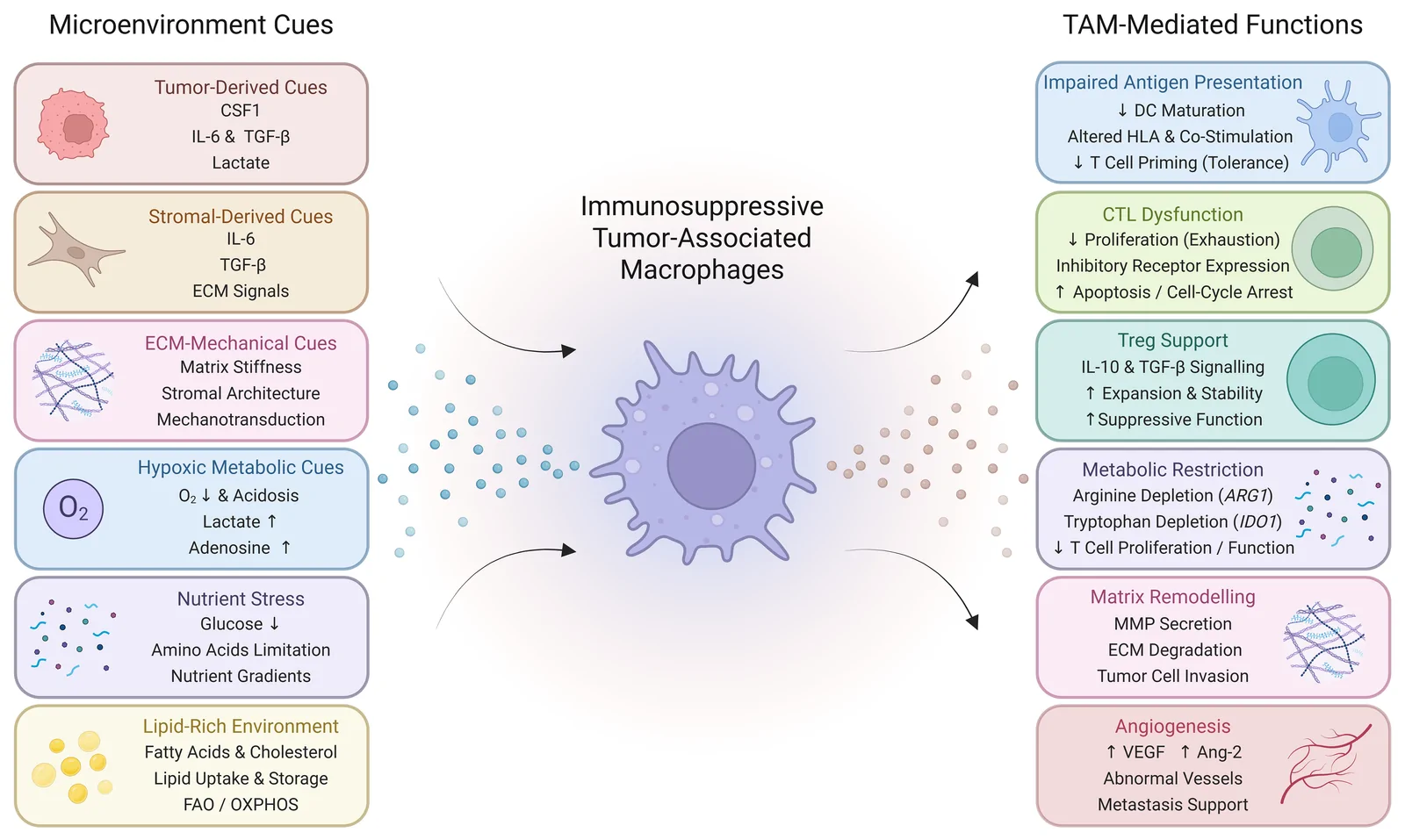
Tumor-associated macrophage plasticity and immunosuppressive function. Schematic representation of tumor-associated macrophages (TAMs) as integrators of microenvironmental cues and mediators of immunosuppressive tumor functions. Tumor-derived, stromal-derived, ECM-mechanical, hypoxic, nutrient-related and lipid-associated cues are shown on the left as inputs that shape TAM behavior. TAM-mediated functions are shown on the right, including impaired antigen presentation, CTL dysfunction, Treg support, metabolic restriction, matrix remodeling and angiogenesis (13, 38, 53).

A high degree of functional plasticity characterizes tumor-associated macrophages (TAMs), enabling continuous adaptation to microenvironmental cues and the acquisition of diverse activation states that support immunosuppression and tissue remodeling (**Figure 2**) (38, 45).

The canonical M1/M2 polarization framework provides a simplified dichotomy that fails to capture the diversity of TAM states observed *in vivo* (41, 50). Transcriptomic analyses, including bulk and single-cell approaches, have revealed a spectrum of activation states, with TAMs co-expressing markers associated with inflammatory, reparative, and regulatory programs (44, 51). For instance, macrophages within hypoxic and stromal tumor niches may simultaneously express genes associated with angiogenesis (e.g., *VEGF*), metabolic suppression (e.g., *ARG1*), and antigen presentation (e.g., *HLA*), traditionally assigned to distinct polarization states (44, 50). These hybrid phenotypes reflect context-dependent integration of signals rather than binary classification (38).

Indeed, TAM-mediated immunosuppression is executed through multiple, partially redundant mechanisms (38, 41). Cytokine production (e.g., IL-10, TGF-β) represents a central suppressive axis, affecting antigen presentation and T-cell function (38, 41). In murine mammary carcinomas, macrophage-derived IL-10 suppressed IL-12 production by intratumoral DCs, limiting CD8+ T-cell-dependent responses to chemotherapy, while IL-10R blockade restored this axis and improved treatment response (52). These cytokines also reinforce macrophage polarization, establishing autocrine loops that stabilize suppressive phenotypes (38, 41).

Metabolic rewiring further underpins TAM function but is strongly context-dependent, with hypoxia, tumor-derived metabolites, and lipid availability shaping distinct suppressive programs (14, 53). Simultaneously, they impose metabolic restrictions on surrounding immune cells through depletion of essential nutrients such as arginine and tryptophan via arginase-1 (ARG1) and indoleamine 2,3-dioxygenase (IDO), thereby restricting T cell proliferation and function (14). Lactate produced by highly glycolytic tumor cells accumulates within the tumor microenvironment and further promotes macrophage polarization toward anti-inflammatory, pro-tumoral phenotypes via hypoxia-inducible factor 1-alpha (HIF-1α)-dependent pathways (54). Lipid metabolism can provide an additional regulatory axis, as fatty-acid uptake and lipid-droplet accumulation were shown to sustain immunosuppressive TAM programming through mTOR-dependent metabolic signaling (55).

Beyond immune modulation, TAMs contribute to tissue remodeling and angiogenesis (45, 56). They secrete matrix metalloproteinases (MMPs) that degrade extracellular matrix components, facilitating tumor invasion (13). Production of pro-angiogenic factors (e.g., VEGF, angiopoietin-2) supports neovascularization, ensuring nutrient supply and enabling metastatic dissemination (38, 56). These functions are tightly integrated with their immunosuppressive roles, as vascular remodeling further influences immune cell trafficking and oxygen gradients (56).

A critical characteristic of TAM biology is the presence of redundancy and compensatory signaling pathways (38, 40). Blocking a single cytokine or receptor often leads to the activation of alternative pathways that preserve the overall suppressive function (40). For example, in the murine 4T1 breast carcinoma model, colony-stimulating factor-1 receptor (CSF1R) inhibition reduced TAM abundance but stimulated CAF production of granulocyte-recruiting chemokines, leading to the accumulation of tumor-promoting polymorphonuclear myeloid-derived suppressor cells (PMN-MDSCs) (57). In a platelet-derived growth factor subunit B (PDGF-B)-driven murine glioma model, prolonged CSF1R inhibition also resulted in acquired resistance mediated by increased macrophage-derived insulin-like growth factor-1 (IGF-1), which activated the IGF-1 receptor - phosphoinositide 3-kinase signaling (IGF-1R-PI3K) in tumor cells and promoted recurrence (58). Beyond pathway redundancy, macrophage plasticity provides an additional mechanism of adaptation to therapeutic pressure (38). In genetically engineered mouse models of pancreatic ductal adenocarcinoma driven by pancreas-specific activation of oncogenic Kras, irradiation induced tumor-cell production of colony-stimulating factor 1 (CSF1) and shifted TAMs toward an immunosuppressive phenotype, thereby suppressing antitumor T-cell responses and limiting radiotherapy efficacy (59). As a result, these compensatory and adaptive responses create network-level resilience that complicates attempts to disrupt TAM-mediated suppression through single-target approaches (40).

Collectively, these features position TAMs not merely as passive responders to suppressive cues but as active drivers of immunosuppression (38, 45). Through sustained feedback interactions with tumor and stromal compartments, as well as modulation of surrounding immune populations, they establish stable regulatory circuits that resist perturbation (41, 45).

### 3.3 Therapeutic targeting of TAMs and its limitations

The central role of TAMs in tumor progression has prompted the development of therapeutic strategies aimed at disrupting their accumulation or function, focusing on three main approaches: depletion, inhibition of recruitment, and blockade of phagocytosis checkpoints (42, 60, 61). While each strategy has demonstrated preclinical efficacy, clinical translation has been limited by adaptive resistance mechanisms, yielding limited clinical benefit as monotherapies (21, 42, 60).

CSF1-CSF1R blockade represents the most extensively studied strategy for macrophage depletion (42, 62). Small-molecule inhibitors and monoclonal antibodies targeting CSF1R have been shown to decrease macrophage survival and reprogram remaining populations, impairing tumor growth in preclinical models (43, 62). However, clinical outcomes have been modest. Residual macrophage populations often persist and may adopt more aggressive or therapy-resistant phenotypes, further contributing to adaptive resistance (40, 42). In parallel, TAM depletion leads to compensatory infiltration of other immunosuppressive cells, including neutrophils and MDSCs (39, 42, 60).

Targeting monocyte recruitment through the CCL2-CCR2 axis represents an alternative strategy to limit TAM accumulation (42, 63). In experimental murine models of breast cancer metastasis, antibody-mediated blockade of CCL2 reduced the recruitment of CCR2-expressing inflammatory monocytes to pulmonary metastasis, inhibited metastatic progression, and prolonged the survival of tumor-bearing mice (63). Nonetheless, clinical translation has been challenging (61). In four syngeneic mouse models of metastatic breast cancer, discontinuation of anti-CCL2 antibody treatment triggered a rebound release of inflammatory monocytes from the bone marrow, promoting IL-6 and VEGF-A-dependent angiogenesis and tumor cell proliferation in pulmonary metastasis, ultimately accelerating metastatic progression (64). This underscores the dynamic nature of myeloid trafficking and highlights a potential limitation of sustained chemokine-axis inhibition, although its direct clinical relevance remains to be established (13, 42).

Phagocytosis checkpoint blockade, particularly targeting the CD47-signal-regulatory protein alpha (SIRPα) axis, seeks to restore macrophage-mediated tumor cell clearance (42, 60, 65). While CD47 blockade enhances phagocytosis and has demonstrated clinical activity, its effects are not restricted to tumor-associated macrophages (42, 66). Widespread expression of CD47 on normal cells has led to on-target toxicities such as anemia (66). Additionally, increased phagocytosis does not necessarily translate into effective antitumor immunity, particularly in immunosuppressive TMEs where antigen presentation and T cell activation remain impaired (42, 60, 67).

Collectively, these approaches underscore a recurring limitation: targeting individual pathways fails to account for the networked and adaptable regulatory nature of TAMs (42, 43, 60). As a result, such strategies often yield transient effects, as redundant signaling networks and compensatory mechanisms can rewire cellular states and restore suppressive function (21, 40). This suggests that TAMs are not defined by individual markers or pathways but by integrated regulatory programs that coordinate their activity (38, 45, 60).

These limitations highlight the need to identify upstream regulators that coordinate multiple suppressive pathways across myeloid subsets. In particular, regulators linking extracellular sensing to intracellular programs represent promising targets (60, 68). Targeting these regulators may disrupt the mechanisms sustaining tumor-associated immune suppression, thereby enabling reprogramming of the myeloid compartment (42, 60, 68).

## 4 Lectin-mediated immune regulation in the TME

### 4.1 Tumor glycosylation shapes the ligand landscape for lectin recognition

Altered glycosylation is increasingly recognized as a hallmark of cancer, with its consequences extending beyond changes in cell-surface composition (1). Alterations in glycan structure, abundance, and presentation reshape the ligand landscape recognized by lectins, a diverse class of glycan-binding proteins that often function as immune receptors (2, 3). Through lectin-mediated recognition, extracellular glycan structures can alter the clustering, membrane distribution and trafficking of cell-surface receptors, thereby shaping signaling programs that regulate immune-cell function (2, 4).

**Figure 3:**
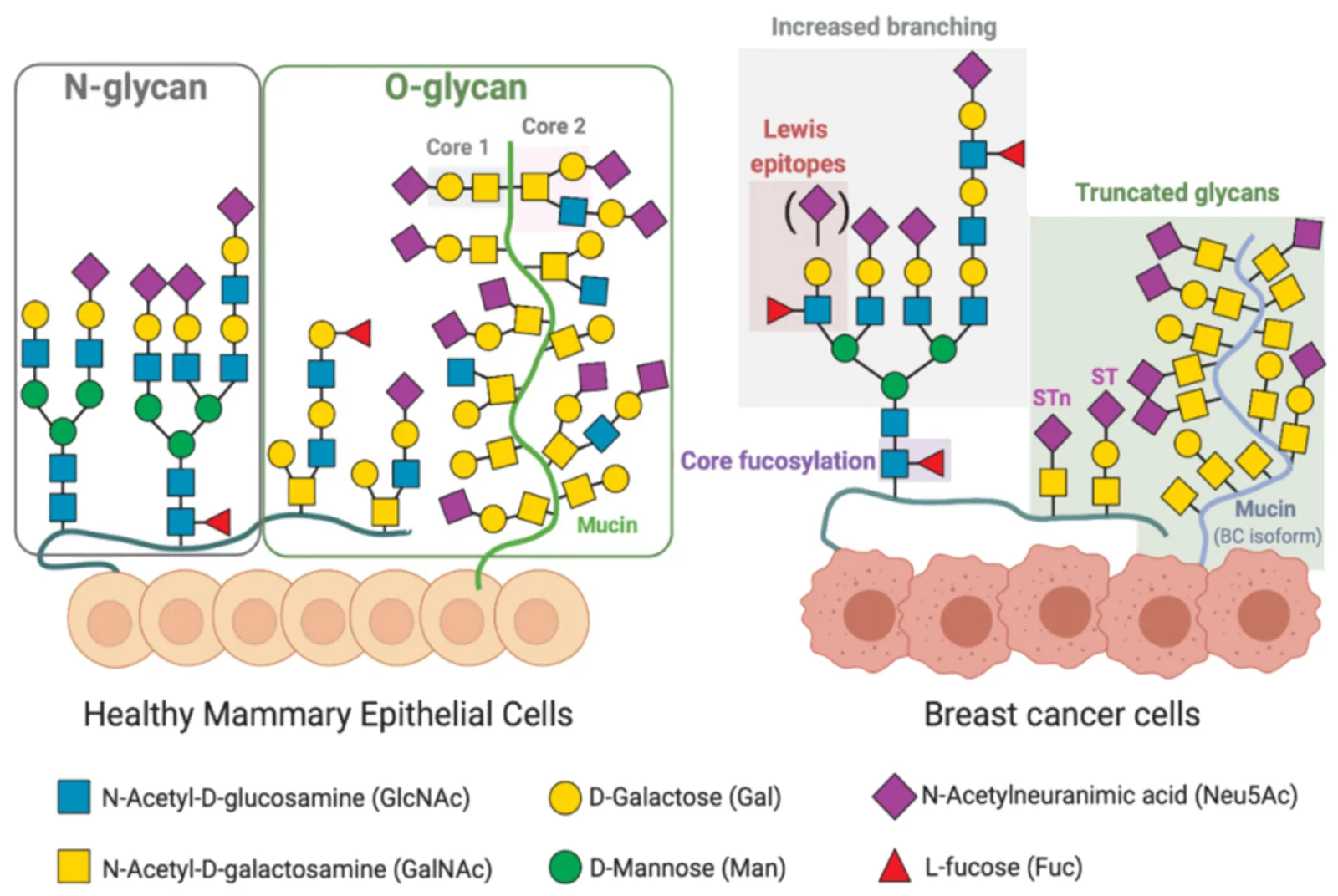
Altered glycosylation on breast cancer cells. Schematic representation of cell-surface glycosylation in healthy mammary epithelial cells and breast cancer cells. Reproduced from Lopes et al. (3) under CC BY 4.0. In healthy mammary epithelial cells, surface glycoproteins display diverse N-glycans, attached to asparagine (Asn) residues through N-acetylglucosamine (GlcNAc), and mucin-type O-glycans, initiated by the attachment of N-acetylgalactosamine (GalNAc) to serine or threonine (Ser/Thr) residues. During breast tumorigenesis, deregulated glycan biosynthesis reshapes the tumor-cell glycocalyx and alters interactions between malignant cells and the surrounding microenvironment. Breast cancer-associated glycosylation changes include increased Lewis antigens (Lewis^a^ and Lewis^x^) and sialylated Lewis antigens (sialyl-Lewis^a^ and sialyl-Lewis^x^), enhanced α1,6-core fucosylation, increased branching of N-glycans, and accumulation of truncated mucin-type O-glycans, including Tn and its sialylated derivative sialyl-Tn. Monosaccharides are represented according to the Symbol Nomenclature for Glycans (SNFG).

The glycan landscape is shaped through the modification of proteins and lipids with structurally diverse glycans (1, 69). Because glycan assembly is not template-encoded but governed by the coordinated activity of glycosylation enzymes, it integrates oncogenic signaling, cellular state, and microenvironmental constraints, such as inflammatory stress and hypoxia, into a context-dependent cell-surface glycome (69). In cancer, this process gives rise to recurrent alterations, including increased β1,6-N-acetylglucosamine (GlcNAc) branching of complex N-glycans, truncation of mucin-type O-glycans, increased sialylation, and altered fucosylation, which collectively reshape the repertoire of glycans displayed at the cell-surface (**Figure 3**) (3).

Mucin-type O-glycosylation is initiated by polypeptide GalNAc-transferases, which add N-acetylgalactosamine (GalNAc) α-linked to serine or threonine residues, generating the Tn antigen structure (69). Tn is converted into core 1 (T antigen) by β1,3-galactosyltransferase 1 (C1GALT1), which can subsequently undergo core 2 branching by β1,6-N-acetylglucosaminyltransferase, and further elongation by diverse glycosyltransferases, generating structurally more complex and diverse O-glycans, elongated by polylactosamine (polyLacNAc) chains capped with fucose, galactose and sialic acid (3, 69). In cancer, impaired core formation or elongation, together with premature terminal sialylation, promote the accumulation and exposure of short O-glycans, including Tn, sialyl-Tn, T, sialyl-T, and disialyl-T (3, 69–71). These aberrantly displayed glycan epitopes constitute prominent tumor-associated carbohydrate antigens (TACAs). For example, ST6GALNAC1 directly sialylates Tn to generate sialyl-Tn, thereby preventing its conversion into more complex O-glycans (72).

N-glycan remodeling provides a distinct regulatory layer. Following the transfer of a common precursor to asparagine residues, N-glycans undergo trimming and remodeling to generate high-mannose, hybrid, and complex structures (69). Cancer-associated alterations can include increased display of high-mannose N-glycans, expanding the ligand repertoire available to mannose-binding lectins, as well as altered branching of complex N-glycans (3, 69). In particular, MGAT5-dependent β1,6-GlcNAc branching of complex N-glycans can influence glycoprotein stability, cell-surface retention and modulate immune-checkpoint function, including PD-L1 glycosylation (1, 73).

Altered terminal glycosylation further diversifies the tumor glycome across both N- and O-glycans. Dysregulated sialyltransferase activity can increase α2,3- and α2,6-linked sialylation and generate cancer-associated epitopes such as sialyl-T and sialyl-Lewis antigens (74–76). Altered fucosyltransferase activity can similarly modify the core fucosylation of N-glycans and the terminal fucosylation of Lewis-family antigens (3, 69). Together, these enzyme-driven alterations define the structural landscape of tumor-associated glycans within the tumor microenvironment.

Recent glycomic and glycoproteomic studies show that these alterations are non-random across tumors and associated with molecular heterogeneity, patient outcome, and therapeutic relevance in specific contexts (77–80). These features define a “glyco-code,” an emergent, context-dependent system in which combinatorial glycan patterns are decoded by lectins to regulate cellular interactions (2, 3). Importantly, this glyco-code is not determined by glycan structure alone. Lectin recognition is also shaped by the glycoprotein or glycolipid on which a glycan is displayed, its density and multivalent presentation, and the membrane and spatial context in which it is encountered. This context dependence is particularly relevant to mucin-type O-glycans, whose site-specific and frequently clustered presentation on densely glycosylated protein domains generates ligand architectures that cannot be inferred from glycan composition alone (81). Accordingly, binding of macrophage galactose-type lectin (MGL) to synthetic mucin glycopeptides increased with both the number of O-linked GalNAc residues and the surface density at which the glycopeptides were presented, directly demonstrating that glycan valency and spatial presentation influence lectin avidity (81, 82). Thus, tumor-associated glycosylation establishes a context-dependent ligand repertoire whose biological effects are determined by the lectin receptors that decode it within the tumor microenvironment.

### 4.2 Lectins as glycan sensors

Three major lectin families dominate the landscape of cancer glyco-immunology, each defined by distinct structural features, glycan specificities, and functional roles (1). C-type lectins (CLRs) comprise a diverse group of receptors classically defined by Ca²^+^-dependent carbohydrate recognition, which regulate processes such as antigen uptake, cell–cell interaction, and immune signaling, with context-dependent roles in both tumor-promoting and tumor-restraining responses (4, 83). I-type lectins, represented by the Siglec family, are sialic acid–binding receptors that predominantly mediate inhibitory signaling and are key sensors of tumor-associated hypersialylation, modulating immune responses primarily in myeloid populations (e.g., monocytes, macrophages, DCs and granulocytes), as well as NK cells, B cells, and selected T cell subsets (84–86). Galectins constitute a third major family, with broad relevance across cancer, and are characterized by β-galactoside recognition. They largely function as soluble or extracellular lectins that organize glycoprotein lattices, thereby influencing receptor clustering, signaling, and cell–cell interactions in tumor, stromal, and immune contexts (87, 88). While these families collectively define the lectin landscape in cancer, their mechanisms of action differ substantially, particularly with respect to their capacity to directly transduce glycan recognition into intracellular signaling.

Lectins recognize the glycan landscape through carbohydrate-recognition domains (CRDs), with binding selectivity determined by monosaccharide composition, linkage, density, multivalency, and presentation (69). Thus, altered glycan structures can be recognized by distinct lectins that act as pattern recognition receptors (PRRs) sensing glycan signatures associated with altered cellular states (4, 89). Within innate immunity, PRRs detect conserved molecular signatures, including pathogen-associated (PAMPs), damage-associated (DAMPs), and self-associated molecular patterns (SAMPs) (4, 89).

Tumor-associated sialoglycans can function as SAMPs by engaging inhibitory Siglecs and dampening immune activation and pro-inflammatory responses (e.g., restraint of tumor-infiltrating T-cell function) (84, 89, 90). In acute myeloid leukemia, this mechanism is illustrated specifically by ST3GAL4-driven α2,3-sialylation increasing the biosynthesis of Siglec-9 ligands, thereby promoting Siglec-9-dependent immune evasion (91).

Self-recognition is shaped by the balance between *cis*-ligands on the same cell surface, which can tonically mask or restrain receptor availability, and *trans*-ligands on neighboring tumor or stromal cells, which can engage lectins across cell–cell interfaces when presented at sufficient density (92, 93). Through this coupling of extracellular glycan recognition to intracellular signaling, lectins directly regulate immune cell function in a context-dependent manner, resulting in either activation or suppression of immune responses, modulation of antigen presentation, or induction of tolerance programs (2, 4, 94). In the tumor microenvironment, these pathways increasingly fulfill the functional logic of immune checkpoints, as glycan–lectin interactions establish inhibitory thresholds that restrain phagocytosis, dampen inflammatory signaling, and promote immunosuppressive myeloid differentiation (2, 90, 95). The CD24-Siglec-10 axis illustrates this function. In co-culture models, CD24 expressed on MCF-7 cancer cells engaged Siglec-10 on donor-derived macrophages to suppress phagocytosis, whereas genetic or antibody-mediated disruption of the axis enhanced tumor-cell engulfment. Consistently, anti-CD24 treatment reduced the growth of established MCF-7 xenografts in immunodeficient mice (95).

Beyond terminal sialic acid glycoconjugate-Siglec interactions, tumor-associated Tn/STn and Lewis-type antigens can be recognized by myeloid C-type lectin receptors such as MGL and dendritic cell-specific intercellular adhesion molecule-3-grabbing non-integrin (DC-SIGN), respectively, thereby influencing how malignant cells are interpreted by antigen-presenting cells (70, 81, 96).

This conceptual framework has led to the definition of glyco-immune checkpoints, in which immune regulation is encoded through glycan–receptor interactions rather than protein–protein binding alone and translated into context-dependent changes in immune cell activation, tolerance, or suppression (2, 84).

### 4.3 Lectin-mediated signal transduction in cancer

In cancer, aberrant glycan signatures engage lectin-mediated recognition pathways and modulate intracellular signaling (84, 85, 90). From a mechanistic standpoint, CLRs and Siglecs, as transmembrane lectins, provide a direct route through which tumor-associated glycans can alter immune-cell state by coupling extracellular glycan recognition to intracellular signaling (2, 4, 84). Signal transduction by these receptors is determined by motifs within their cytoplasmic domains or by their association with signaling adaptor proteins (4, 84).

Activating CLRs may signal through an intrinsic hemi-immunoreceptor tyrosine-based activation motif (hemITAM), which contains a single YxxL motif, as exemplified by Dectin-1, or through associated adaptors bearing a canonical immunoreceptor tyrosine-based activation motif (ITAM), which contains two YxxL/I motifs (4, 97). These adaptors include the Fc receptor γ-chain (FcRγ) or DNAX activation protein of 12 kDa (DAP12) (98). Following receptor engagement, Src-family kinases phosphorylate these motifs, enabling spleen tyrosine kinase (SYK) recruitment and activation of downstream pathways, including CARD9-BCL10-MALT1-dependent nuclear factor kappa-light-chain-enhancer of activated B cells (NF-κB) signaling, mitogen-activated protein kinase (MAPK), PI3K, and phospholipase Cγ (PLCγ)-dependent calcium signaling (4, 99).

**Figure 4:**
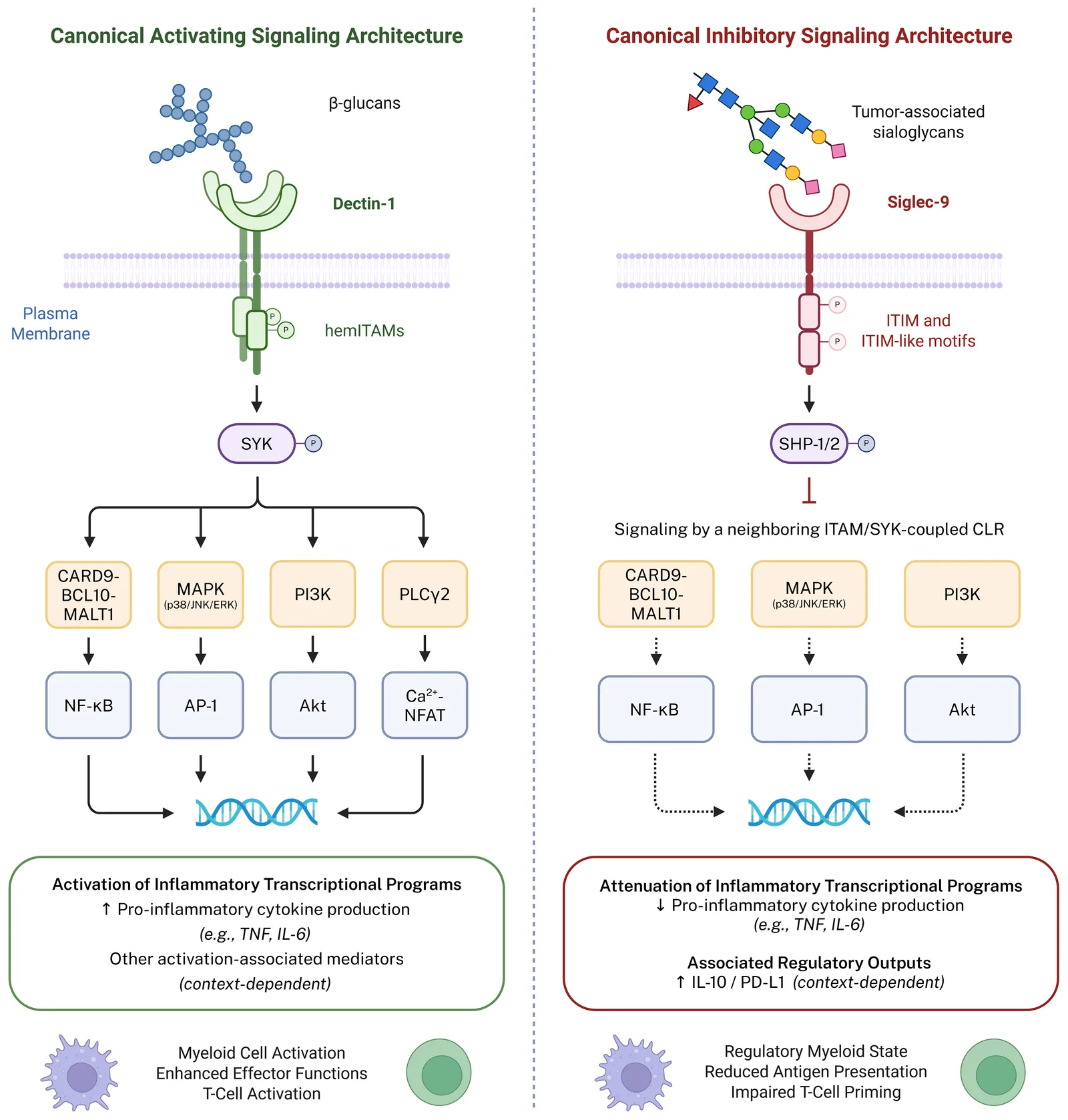
Representative signaling architectures of myeloid transmembrane lectins. Dectin-1 illustrates a canonical hemITAM–SYK-coupled activating architecture, whereas Siglec-9 illustrates canonical inhibitory signaling through phosphorylated ITIM and ITIM-like motifs and recruitment of SHP-1/2, which can attenuate signaling initiated by neighboring activating receptors. The pathways shown are illustrative and do not imply that the receptors are functional opposites. Biological outcomes vary with ligand identity and presentation, receptor clustering and crosstalk, cellular state, and tumor context.

Inhibitory CLRs bearing immunoreceptor tyrosine-based inhibitory motifs (ITIMs) instead recruit Src homology 2 domain-containing phosphatases 1 and 2 (SHP-1 and SHP-2) following motif phosphorylation by Src-family kinases, thereby attenuating activating signals (4, 100). Receptors lacking canonical ITAM or ITIM motifs, including DC-SIGN, can modulate signaling through alternative routes such as Raf-1 (101). Many CD33-related Siglecs similarly contain cytoplasmic ITIM and ITIM-like motifs that recruit SHP-1/2, although this architecture is not universal (84, 102). Siglec-15, for instance, associates with DAP12 and signals through SYK (103). Thus, lectin signaling outcomes are determined not by glycan specificity or family membership alone, but by receptor architecture, adaptor usage, and cellular context (**Figure 4**) (4, 84).

These receptor-coupled signaling architectures enable transmembrane lectins to regulate phagocytosis, antigen processing, cytokine production, metabolic wiring, and checkpoint expression (95, 104–106). This signaling capacity further underpins their therapeutic potential, as targeting lectin-mediated pathways can reshape immune responses and enhance sensitivity to immunotherapy (107, 108).

Galectins remain highly relevant to cancer immunity, but they operate through a distinct mechanistic logic (87, 88). They are not transmembrane receptors and instead function predominantly as soluble or matrix-associated glycan binders in the extracellular compartment, where they reorganize glycoprotein topology, receptor residency, and cell–cell or cell–matrix interactions through multivalent lattice formation (87). This mechanism is illustrated by galectin-3, which can retain T-cell receptors within glycoprotein lattices on tumor-infiltrating lymphocytes, restricting receptor mobility and impairing interferon-γ secretion (109). In tumor-associated endothelial cells, galectin-1 binds complex N-glycans on vascular endothelial growth factor receptor 2 (VEGFR2), prolonging its surface residency and sustaining angiogenic signaling in tumors resistant to anti-VEGF therapy (110).

While the biological impact of galectins is substantial across tumor, stromal, and immune compartments, their effects are mediated through extracellular network organization and glycoprotein lattice formation rather than discrete receptor-associated signaling modules (87, 88). By contrast, transmembrane lectins directly translate glycan recognition into intracellular immune-cell signaling, linking these extracellular cues to defined myeloid cell states and providing potential targets for therapeutic modulation (2, 87).

### 4.4 Integration of lectin signaling in immunosuppressive programs

Glycan-driven immunosuppression cannot be explained by single lectin-ligand pairs considered in isolation (2, 3). The same suppressive niche can present Tn/STn-bearing mucins, hypersialylated glycoconjugates, fucosylated Lewis antigens, and stromal glycans at the same time, while the same cell can express several lectins capable of reading those inputs (2). This architecture creates redundancy but it also creates combinatorial control. In pancreatic cancer, tumor and stromal compartments display distinct sialylated ligand repertoires. Tumor-cell sialic acids promote monocyte-to-macrophage differentiation through Siglec-7 and Siglec-9, whereas cancer-associated fibroblast-derived sialoglycans engage Siglec-7, -9, -10, and -15 on myeloid cells and collectively drive TAM differentiation (86, 111). Other Siglec axes reinforce complementary suppressive functions: CD24-Siglec-10 signaling inhibits macrophage-mediated phagocytosis, while Siglec-15 suppresses antigen-specific T-cell responses (95, 112). Additionally, Siglec-9-associated TAM states have been linked to impaired T-cell priming and poor response to PD-1 blockade (107). These observations indicate that lectin function in the tumor microenvironment emerges from coordinated interactions between multiple receptors and glycan ligands rather than from isolated receptor-ligand pairs.

These lectin networks intersect with several major axes already known to shape suppressive myeloid cells: cytokine signaling, metabolic adaptation, and checkpoint biology (2, 43). Tumor-associated sialylation can promote immunosuppressive macrophage differentiation through Siglec-7 and Siglec-9, including increased IL-10 and PD-L1 expression (111, 113). However, these outputs should not be interpreted as arising from a uniform ITIM-dependent inhibitory mechanism: in the MUC1-ST–Siglec-9 axis, macrophage reprogramming is associated with calcium flux and MAPK/ERK kinase (MEK)–extracellular signal-regulated kinase (ERK) activation rather than classical SHP-1/2 signaling (94). In parallel, MGL binding by terminal GalNAc-containing ligands rewires dendritic cell metabolism toward reduced glycolytic activity and a less inflammatory state (105). Dectin-1-dependent circuits further illustrate the contextual plasticity of CLR signaling, with Dectin-1 stimulation producing opposing effects in murine melanoma and colorectal cancer models (108). Together, these examples underscore that the biological outcome depends on receptor repertoire, ligand context, and tissue ecology. Accordingly, CLRs and Siglecs emerge as the transmembrane lectin families that can act as upstream coordinators translating the glycan architecture of the tumor microenvironment into context-dependent myeloid states, including stable immunosuppressive programs (2, 86, 90).

## 5 Transmembrane myeloid lectins in cancer immunity

### 5.1 C-type lectin receptors

CLRs constitute a structurally defined group within the receptors containing a C-type lectin-like domain (CTLD) (4, 114, 115). While historically described as Ca²^+^-dependent carbohydrate-binding receptors, this definition does not uniformly apply across the family. The C-type lectin-like domain (CTLD) represents a conserved structural fold, characterized by a compact double-loop architecture stabilized by disulfide bonds, rather than a strict functional annotation (114). Accordingly, ligand recognition in classical carbohydrate-binding CLRs is often Ca²^+^-dependent and influenced by conserved motifs such as EPN (Glu-Pro-Asn) and QPD (Gln-Pro-Asp), which preferentially recognize mannose and fucose residues or galactose and N-acetylgalactosamine (GalNAc), respectively (114, 115). However, several receptors deviate from these canonical rules and have evolved Ca²^+^-independent configurations that enable recognition of non-carbohydrate ligands (4, 116). Receptors such as Dectin-1 (CLEC7A) and CLEC9A bind proteins and endogenous ligands exposed during cell stress or death, illustrating that CLR function extends beyond glycan recognition alone (4, 104, 117).

Within the tumor microenvironment, CLRs function as transmembrane sensors that integrate extracellular cues derived from TACAs (SAMPs) and necrotic tumor debris (DAMPs) into intracellular signaling programs (2, 4). Aberrant glycosylation generates diverse tumor-associated ligands for CLRs, including truncated mucin-type O-glycans (Tn and sTn), terminal GalNAc-containing motifs such as LacdiNAc on N- and O-glycans, and fucosylated Lewis antigens (3, 70). MGL recognizes terminal GalNAc-containing structures, whereas DC-SIGN binds specific tumor-associated Lewis-antigens, thereby modulating antigen-presenting-cell differentiation, macrophage function, and cytokine responses (3, 70, 96, 105). In parallel, tumor-associated cell death releases endogenous DAMPs, including exposed cytoskeletal components and intracellular metabolites, which are sensed by receptors such as Mincle and members of the Dectin family (4, 104, 117, 118). CLEC-1 (CLEC1A) provides another example of a death-sensing CLR, limiting dendritic-cell activation and cDC1-mediated cross-presentation of dead-cell-associated antigens. Its knockout or therapeutic blockade enhanced antitumor immune responses in preclinical models, supporting CLEC-1 as an emerging myeloid immune-checkpoint target (119). Through this coupling, CLRs regulate antigen handling, inflammatory signaling, and the establishment of suppressive or tolerogenic myeloid states, positioning them as integrators of tumor-derived signals rather than strictly glycan-restricted receptors (2, 4, 120).

Given the structural diversity of the CTLD superfamily and the lack of strict correspondence between phylogenetic classification and immune function, myeloid CLRs can thus be organized according to their intracellular signaling architecture and dominant functional output rather than formal subfamily nomenclature (4, 116, 120).

A first class comprises activating receptors, including Dectin-1 (CLEC7A), Dectin-2 (CLEC6A), Mincle (CLEC4E), and MCL (CLEC4D), which signal through hemITAM motifs or FcRγ-associated ITAM motifs (immunoreceptor tyrosine-based activation motifs), leading to SYK recruitment and activation of the CARD9–BCL10–MALT1 axis, with downstream engagement of NF-κB and inflammatory transcriptional programs (4, 116, 120). In cancer, Dectin-2 and MCL/CLEC4D were shown to support Kupffer-cell phagocytosis and suppression of liver metastasis in murine models, while subsequent work identified an ERMAP–galectin-9–Dectin-2 bridging complex as a glycosylation-dependent “eat-me” signal promoting Kupffer-cell clearance of cancer cells (121, 122).

A second class comprises inhibitory receptors, such as DCIR (CLEC4A) and MICL (CLEC12A), which contain ITIM motifs (immunoreceptor tyrosine-based inhibitory motifs) that recruit phosphatases such as SHP-1 and SHP-2, thereby attenuating activation signals and raising the threshold for immune activation (4, 100, 123). Beyond its established role in regulating inflammatory thresholds, CLEC12A has recently emerged in glioblastoma as a lectin-network hub, together with ASGR2, associated with immunosuppressive myeloid populations (124). By contrast, the role of DCIR remains less established and appears context-dependent. Evidence from colorectal cancer illustrates this variability. In a colitis-associated carcinogenesis model, DCIR deficiency or blockade restrained tumor development through enhanced GM-CSF-STAT5 signaling and reduced accumulation of suppressive myeloid cells. Conversely, in an orthotopic syngeneic tumor model, DCIR deficiency promoted tumor growth, altered TAM phenotypes, and reduced effector T-cell activation (123, 125).

Beyond these motif-defined receptors, several myeloid CLRs lack classical ITAM or ITIM motifs but should not be treated as a single residual category. MGL (CLEC10A) and DC-SIGN (CD209) are better considered non-canonical signaling-modulatory CLRs: MGL ligation by terminal GalNAc-bearing ligands modulates dendritic-cell inflammatory and metabolic programming, including reduced glycolytic activity and promotion of IL-10-associated regulatory programs, whereas DC-SIGN shapes inflammatory signaling through Raf-1-dependent crosstalk with TLR-NF-κB pathways (96, 101, 105). More recently, Lewis^X^ ligation of DC-SIGN in colorectal TAMs was shown to induce BCL-3-dependent PD-L1 expression, directly linking receptor engagement to an immune-checkpoint program (126). By contrast, the mannose receptor CD206/MRC1 functions predominantly as an endocytic and scavenger receptor, regulating ligand internalization and antigen routing rather than signaling through a canonical intracellular motif (120). In TAMs, these functions can indirectly influence tissue remodeling, such as CD206-mediated uptake and lysosomal degradation of collagen fragments by TAMs that contribute to extracellular-matrix turnover in murine tumor models. Its functional effects are also context-dependent, as CD206 engagement reprogrammed CD206-high M2-like macrophages toward an antitumor phenotype but had little effect on M1-like macrophages (127, 128).

At the functional level, these distinct receptor mechanisms converge on a limited set of outputs that shape myeloid cell behavior in the tumor microenvironment (2, 4). These include antigen handling and routing, exemplified by CLEC9A-mediated cross-presentation of cell-associated antigens and consequent CD8^+^ T-cell priming; signaling-modulatory and endocytic CLRs additionally regulate inflammatory thresholds, cellular metabolism, scavenging and extracellular matrix turnover (96, 104, 105, 127, 129). These functions are not independent but operate in an integrated manner to control antigen availability and myeloid activation states, positioning CLRs as upstream regulators that translate extracellular cues into context-dependent immune responses within the TME (2–4).

While this framework captures the core regulatory logic of CLR-mediated recognition and signaling, it represents only a subset of a broader repertoire of CTLD-containing receptors expressed across myeloid populations. A more comprehensive overview of myeloid lectins, including additional receptors with modulatory or context-dependent roles in cancer that extend beyond the canonical CLR subsets, and emerging therapeutic targeting strategies, is provided in **Table 1**. This overview integrates ligand specificity, signaling features, and functional associations to contextualize the diversity of lectin-mediated regulation within the tumor microenvironment.

**Table 1:** Selected C-type lectin receptors and related CTLD-containing transmembrane receptors in myeloid regulation and cancer immunity.

Receptor	Subfamily/group	Ligands & specificity	Expression profile	Signaling motif	Mechanistic & functional output	Evidence & relevance in cancer	Therapeutic targeting/modulation	Relevance to TAMs	Refs
MGL (CLEC10A)	Type II CLR	GalNAc-terminated motifs, including Tn, sTn, and LacdiNAc, often displayed on mucin-type O-glycans such as MUC1	DCs, macrophages	Endocytic	Promotes IL-10-associated regulatory programming and reduces glycolytic activity in human monocyte-derived DCs; mediates uptake and processing of GalNAc-bearing ligands	High tumor MGL-ligand expression is associated with poor disease-free survival in stage III colorectal cancer	Glycan–MGL axis targeted by glycomimetics and antibodies (preclinical)	Potential contributor to regulatory myeloid states; direct TAM-polarizing causality in cancer remains incompletely defined	(3, 81, 105, 115, 157–159)
DC-SIGN (CD209)	Type II CLR	Lewis antigens (Le^a^, Le^b^, Le^x^, Le^y^), high-mannose glycans, CEA, clusterin	DCs, macrophages	Endocytic/signaling crosstalk	Modulates TLR signaling through Raf-1-dependent crosstalk; in CRC TAMs, Lewis^X^ ligation induces BCL-3-dependent PD-L1 expression	DC-SIGN^high^ TAMs are associated with an immune-evasive PD-L1^high^ program and poorer outcome in CD8-high colorectal cancer	Glycomimetic and multivalent glycodendrimer antagonists have been developed to block DC-SIGN–glycan interactions; cancer relevance remains mainly preclinical, with potential use in combinatorial immunotherapy.	Defines a context-dependent regulatory TAM population in colorectal cancer	(96, 101, 115, 126, 157, 160)
CD206 (MRC1)	Mannose receptor	Mannose, fucose, GlcNAc-containing glycans	Macrophages, DCs	Endocytic	Antigen scavenging, endocytosis, ECM remodeling, macrophage polarization; can support CXCL9-mediated T cell recruitment in specific contexts	Strongly associated with TAM infiltration and tumor progression	Targeted by peptides (e.g., RP-182) to induce TAM reprogramming and phagocytosis	Canonical TAM marker with regulatory and remodeling functions	(115, 120, 127, 129)
Dectin-1 (CLEC7A)	Dectin-1 cluster	β-glucans, endogenous ligands (e.g., galectin-9)	Broad myeloid	hemITAM	SYK activation, ROS production, NF-κB signaling, cytokine release (IL-6, TNF); context-dependent inflammatory or suppressive effects	Dual role in cancer: Dectin-1 stimulation with curdlan reduced B16-F10 melanoma growth but promoted CT26 colorectal tumor growth in murine models	Agonists (e.g., curdlan) can enhance anti-tumor immunity in selected contexts, but therapeutic effects are tumor-dependent	Modulates TAM activation and plasticity	(108, 115, 120, 161–163)
Dectin-2 (CLEC6A)	Dectin-2 cluster	High-mannose glycans, galectin-9-associated ERMAP bridging complex described in cancer	Macrophages, DCs	FcRγ (ITAM)	FcRγ–SYK–CARD9 signaling; promotes inflammatory responses and, in Kupffer cells, phagocytic clearance of cancer cells	Dectin-2-dependent Kupffer-cell phagocytosis restrains liver metastasis in murine models; an ERMAP–galectin-9–Dectin-2 complex functions as a glycosylation-dependent pro-phagocytic axis	Agonistic targeting shown to reprogram TAMs in preclinical models	Supports antitumor phagocytosis in liver-resident Kupffer cells; effects are likely tissue- and context-dependent	(4, 98, 115, 120, 121)
Mincle (CLEC4E)	Dectin-2 cluster	SAP130, β-glucosylceramide, cholesterol crystals, DAMPs	Macrophages, neutrophils	FcRγ (ITAM)	Induces inflammatory cytokines (TNF, IL-6), sterile inflammation, sensing of necrotic cells; promotes tumor-supportive inflammation	Promotes tumor progression (e.g., pancreatic cancer) via necrosis sensing	Targeting under exploration; potential for modulating tumor inflammation	Drives pro-tumoral inflammation and TAM activation in necrotic niches	(115, 118, 120, 164)
CLEC-1 (CLEC1A)	Dectin-1 cluster	TRIM21; ligands exposed by cells undergoing programmed necrosis	Macrophages, DCs	No canonical ITAM/ITIM assignment; receptor-proximal signaling remains incompletely defined	Limits cDC1 cross-presentation of dead-cell-associated antigens and restrains DC activation and downstream T-cell responses	CLEC-1 loss reduces immunosuppressive myeloid-cell accumulation and enhances antitumor immune responses in murine tumor models	Antagonist anti-CLEC-1 antibodies; preclinical antitumor activity, including combination with chemotherapy	Expressed in tumor-infiltrating myeloid cells and contributes to an immunosuppressive TME	(115, 119)
CLEC4D	Dectin-2 cluster	Microbial lipids, endogenous ligands (poorly defined)	Myeloid cells	FcRγ (ITAM)	SYK-dependent inflammatory signaling; contributes to cytokine production and myeloid activation	Cooperates with Dectin-2 in Kupffer-cell phagocytosis and suppression of liver metastasis in murine models	Not yet well developed therapeutically	Supports antitumor phagocytic activity of liver-resident macrophages	(4, 83, 115, 120, 121, 165)
CLEC9A (DNGR-1)	Dectin-1 cluster	F-actin (necrotic cells)	cDC1	hemITAM	Regulates endosomal routing, enables cross-presentation of dead-cell antigens on MHC I, supports CD8^+^ T cell priming without strong inflammation	Critical for anti-tumor immunity via T cell priming	Target for vaccine strategies and antigen delivery platforms	Indirect TAM relevance via antigen presentation axis	(4, 104, 115, 117, 166)
DCIR (CLEC4A)	Dectin-2 cluster	Glycans (low affinity)	DCs, macrophages	ITIM	Recruits SHP-1/2, suppresses activation, modulates cytokine production (GM-CSF, IL-6), limits inflammatory signaling	Context-dependent in colorectal cancer: DCIR loss/blockade restrained colitis-associated tumorigenesis in one model, whereas DCIR deficiency promoted tumor growth in an orthotopic model	Blockade showed antitumor activity in one preclinical CRC model, but opposing genetic evidence limits generalization	Modulates suppressive myeloid accumulation and TAM phenotypes in a model-dependent manner	(4, 115, 123, 125)
CLEC12A (MICL)	Dectin-1 cluster	DAMPs (e.g., uric acid crystals)	Monocytes, macrophages	ITIM	Inhibitory signaling, suppression of sterile inflammation, regulation of activation thresholds	Highly expressed in AML; marker of leukemic stem cells; differential co-expression hub in glioblastoma and expressed across tumor-infiltrating myeloid populations with poorer prognosis	Target of CAR-T cells, bispecific antibodies, immunocytokines (e.g., IL-15 fusions)	Controls inflammatory thresholds in myeloid cells; emerging association with immunosuppressive myeloid networks in glioblastoma	(100, 115, 120, 124)
CLEC2B	CTLD-containing receptor	NKp80	Immune cells	No established CLEC2B-intrinsic ITAM/ITIM signaling motif	Functions principally as the cell-surface ligand for NKp80; the atypical hemITAM is located in NKp80, not CLEC2B/AICL	Limited data in cancer	Not yet targeted	Potential regulatory role in immune communication	(115, 167, 168)
CD72	ITIM receptor (CTLD-related)	Semaphorin ligands (e.g., Sema4D)	B cells, some myeloid	ITIM	SHP-1 recruitment, inhibitory signaling, regulation of immune activation	Implicated in immune regulation; emerging relevance in tumor immunity	Potential immunomodulatory target (early stage)	May contribute to suppressive signaling networks	(114, 115, 169, 170)
SELL (CD62L)	Selectin (lectin domain)	Sialylated glycans (e.g., PNAd)	Leukocytes	Non-ITAM/ITIM	Mediates leukocyte trafficking, homing, adhesion; regulates immune cell localization	Indirect role in tumor immunity via immune infiltration	Targeted in inflammation; indirect cancer relevance	Controls immune cell recruitment to tumor sites	(114, 115, 157, 171, 172)

The table summarizes receptor classification, ligand specificity, expression, signaling, functional output, cancer relevance, therapeutic modulation, and relevance to TAMs. Only selected receptors are included; ligand recognition and functional consequences may vary with carrier context, density, multivalency, and spatial presentation.

### 5.2 I-type lectins (e.g., Siglecs)

I-type lectins fall within the immunoglobulin superfamily as a subset of proteins characterized by the presence of Ig-like domains (92, 115). In the context of cancer immunity, this glycan-recognition function is predominantly mediated by the sialic acid-binding immunoglobulin-like lectins (Siglecs), which constitute the principal class of transmembrane I-type lectins specialized in decoding sialylated glycoconjugates within the TME (85, 86, 92, 102).

Structurally, Siglecs are characterized by a single N-terminal V-set Ig-like domain that contains the carbohydrate-recognition domain (CRD), followed by a variable number of C2-set Ig-like domains that project the receptor away from the plasma membrane (92). Ligand binding is mediated by a conserved arginine residue within the V-set domain that enables recognition of sialic acids (92). Binding specificity is further shaped by a highly variable C-C’ loop of this V-set domain, which determines preference based on the sialic acid linkage (α2-3, α2-6, or α2-8) and the underlying spatial arrangement of the carrier glycoprotein or glycolipid (86, 92).

These receptors are commonly divided into two evolutionary groups: conserved Siglecs and CD33-related Siglecs - named after CD33 (Siglec-3), the prototypical member of this group (84, 92). Conserved Siglecs, including Siglec-1, Siglec-2, Siglec-4, and Siglec-15, show clearer orthology across mammals and tend to have more stable structural and functional conservation (84, 92). In contrast, CD33-related Siglecs have expanded rapidly during primate evolution and display greater variability in ligand specificity, expression, and signaling properties (84, 92).

Siglec expression is predominantly enriched across myeloid populations, including monocytes, macrophages, dendritic cells, and granulocytes, with receptor-specific expression patterns that shape functional specialization (86, 92, 120). Accordingly, the canonical Siglec architecture suppresses downstream transcriptional programs and raises the threshold for immune cell activation (84, 85, 92). In macrophages, this inhibitory signaling axis can interfere with cytoskeletal rearrangements required for phagocytosis, while in dendritic cells it limits antigen presentation and costimulatory signaling, establishing a signaling framework that constrains myeloid activation (85, 86, 95). Consistent with this canonical architecture, selective co-engagement of Siglec-9 and Fcγ receptors in a human monocytic cell model recruited inhibitory phosphatases and suppressed FcγR activation (130). However, this ITIM–phosphatase framework represents the canonical signaling logic of many Siglecs rather than a universal outcome of Siglec engagement, as ligand- and carrier-specific interactions can generate non-canonical signaling outputs, with MUC1-ST engagement of Siglec-9 on primary monocytes and macrophages inducing a non-canonical calcium-dependent MEK–ERK signaling response rather than SHP-1/2 activation (94).

This inhibitory framework is sustained by widespread hypersialylation of tumor and stromal glycoconjugates, driven by altered sialyltransferase activity and enriched sialylated carriers such as mucins, which promote dense and multivalent presentation of sialoglycans at the cell surface (74–76, 85). This organization generates a ligand-rich interface that facilitates Siglec engagement on infiltrating myeloid cells (85, 111, 113). In murine tumor models, sialoglycan engagement of Siglec-E promoted CCL2 production as a downstream mediator of MDSC-dependent T-cell suppression, with myeloid Siglec-E deficiency reducing suppressive activity and prolonging survival. Consistent with a related mechanism in humans, primary tumor-MDSCs showed sialoglycan-dependent CCL2 secretion, although the analog human Siglec remain unresolved (106). These findings further illustrate how tumor-associated sialoglycans can function as SAMPs that suppress myeloid activation and reinforce tolerogenic programs, establishing a central axis of glyco-immune checkpoint regulation (2, 84, 86).

In cancer, these mechanisms are illustrated in distinct Siglec–ligand axes. Siglec-7 is expressed by NK cells and subsets of monocytes and macrophages, and recognizes tumor-associated sialoglycans, including α2,8-disialylated motifs, gangliosides such as GD2/GD3, and branched α2,6-sialylated structures (86, 92, 111). Together with Siglec-9, it has been implicated in sialic acid–dependent macrophage differentiation and suppressive myeloid programming in tumor contexts, particularly in pancreatic cancer models (86, 111). The interaction between CD24 and Siglec-10 functions as a dominant “don’t eat me” signal, inhibiting macrophage-mediated phagocytosis independently of Fc receptor signaling, similarly to the CD47–SIRPα checkpoint (95, 131). More recently, sialylated integrin α3β1 was identified as a Siglec-10 ligand in pancreatic cancer and disruption of this interaction enhanced macrophage phagocytosis and reduced tumor growth in both a human macrophage xenograft and a transgenic mouse models (132).

A mechanistically distinct example is provided by MUC1-ST–Siglec-9 signaling (94). Hypersialylated MUC1 can independently drive monocyte-to-macrophage differentiation toward a pathogenic TAM-like phenotype (increased expression of IDO, CD163, CD206, PD-L1) associated with impaired CD8^+^ T cell proliferation, reduced phagocytic efficiency, basement membrane disruption, and stromal macrophage enrichment in breast cancer (133).

In contrast, Siglec-15, which signals through the ITAM-bearing adaptor DAP12 rather than ITIM-mediated inhibition, has emerged as a distinct myeloid checkpoint that suppresses antigen-specific T cell responses (103, 112, 134). Its expression on tumor and tumor-infiltrating myeloid cells can occur independently of PD-L1, supporting Siglec-15 as an alternative immunosuppressive axis in PD-L1-low or -negative tumors (112). Therapeutically, an anti-Siglec-15–GM-CSF chimera reprogrammed immunosuppressive TAMs, enhanced macrophage antigen presentation and T-cell activation, and suppressed colorectal tumor growth (135). However, its ligand biology remains incompletely resolved, with evidence supporting sialoglycan-dependent recognition beyond a simple sialyl-Tn-restricted model (134, 136).

While this framework captures the core regulatory logic of Siglec-mediated signaling, it represents only a subset of a broader and heterogeneous receptor family with context-dependent roles in cancer. A comprehensive overview of I-type lectins, including Siglec family members and additional immunoglobulin superfamily proteins with lectin-like domains (e.g., ICAM1, CD83), is provided in **Table 2**. This overview integrates ligand specificity, expression patterns, signaling features, and functional relevance to contextualize the diversity of lectin-domain–containing receptors in tumor immunity, including those that contribute indirectly to glycan-dependent regulation through adhesion or immunomodulatory functions.

**Table 2:** Selected I-type lectins and related immunoglobulin-superfamily receptors in myeloid regulation and cancer immunity.

Receptor	Subfamily/group	Ligands & specificity	Expression profile	Signaling motif	Mechanistic & functional output	Evidence & relevance in cancer	Therapeutic targeting/modulation	Relevance to TAMs	Refs
Siglec-1 (CD169)	Conserved Siglec	Predominantly α2,3-linked sialoglycans and selected α2,8-containing gangliosides; sialylated tumor vesicles/exosomes	Tissue-resident macrophages (CD169^+^ subsets)	No canonical ITIM/ITAM	Endocytosis and capture of sialylated particles; antigen routing and cross-presentation support	Associated with antigen capture and immune activation in some tumor contexts; prognostic value is context-dependent	Explored for antigen delivery and vaccination strategies	Context-dependent macrophage subset associated with antigen capture and cross-presentation in some settings, but with immunosuppressive functions in others.	(92, 115, 120, 173–175)
Siglec-7	CD33-related Siglec	α2,8-disialylated motifs; gangliosides such as GD2/GD3; branched α2,6-sialylated glycans	NK cells, monocytes, macrophages	ITIM/ITIM-like	SHP-1/2 recruitment; inhibition of cytotoxicity and myeloid activation; dampening of pro-inflammatory signaling	Implicated in tumors enriched in gangliosides (e.g., melanoma); contributes to immune evasion	Targeting gangliosides or Siglec–glycan interactions under investigation	Modulates macrophage activation and differentiation toward suppressive phenotypes	(86, 92, 111, 115, 120, 176)
Siglec-9	CD33-related Siglec	α2-3/α2–6 sialylated glycans; sialyl-Lewis X; MUC1-ST	Monocytes, macrophages, DCs, neutrophils	ITIM/ITIM-like	Canonical ITIM/ITIM-like signaling can recruit SHP-1/2 and suppress activating-receptor signaling, including FcγR activation; MUC1-ST engagement can induce non-canonical Ca²^+^–MEK/ERK signaling; impaired DC maturation, induction of IL-10/PD-L1, and suppression of T cell priming	MUC1-ST–Siglec-9 axis promotes TAM-like polarization through non-canonical signaling; Siglec-9-associated myeloid states correlate with immune suppression and poor prognosis in selected cancers	Siglec-9 blockade and tumor desialylation strategies explored preclinically	Contributes to monocyte-to-macrophage/TAM-like differentiation and immunosuppressive programming in selected tumor contexts	(3, 92, 94, 107, 111, 115, 120, 130, 176)
Siglec-10	CD33-related Siglec	α2-3/α2–6 sialylated glycans; CD24; CD52; sialylated integrin α3β1	Macrophages, monocytes, DCs, B cells	ITIM/ITIM-like; Grb2-associated motif	SHP-1/2-mediated inhibition of cytoskeletal remodeling; blockade of phagocytosis; suppression of inflammatory signaling	CD24–Siglec-10 axis acts as a dominant glyco-immune “don’t eat me” checkpoint in multiple cancers (e.g., TNBC, ovarian); in PDAC, sialylated α3β1–Siglec-10 signaling suppresses macrophage-mediated tumor-cell phagocytosis	CD24/Siglec-10 blockade and disruption of α3β1–Siglec-10 interactions restore macrophage phagocytosis in preclinical models	Key anti-phagocytic checkpoint in TAMs; parallels CD47–SIRPα axis	(3, 86, 92, 95, 115, 120, 132)
Siglec-15	Conserved Siglec	Sialoglycans, including α2-6-linked sialylated glycans; sialyl-Tn proposed but not consistently validated as dominant cell-surface ligand	TAMs, osteoclasts, tumor cells (NSCLC, colon, thyroid)	DAP12-associated ITAM	Associates with DAP12/DAP10-adaptor signaling; activates SYK/MAPK-associated pathways; promotes immunosuppressive outputs, including inhibition of antigen-specific T cell responses; TGF-β induction appears context-dependent	Expressed in subsets of PD-L1–negative tumors; implicated as a non-redundant immune-suppressive checkpoint; ligand specificity remains incompletely resolved and may extend beyond sialyl-Tn	Antibody-mediated Siglec-15 blockade has shown preclinical antitumor activity; an anti-Siglec-15–GM-CSF chimera showed preclinical TAM reprogramming and antitumor activity	Highly expressed in TAMs; suppresses effector T cell responses	(3, 103, 112, 115, 120, 134–136)
ICAM1 (CD54)	Ig-superfamily adhesion molecule (non-canonical lectin-associated)	Binds LFA-1 (CD11a/CD18) and Mac-1; glycosylation modulates binding and immune interactions	Endothelial cells, tumor cells, leukocytes, activated myeloid cells	No ITIM/ITAM	Regulates leukocyte adhesion, trafficking, and immune synapse formation; NF-κB–dependent expression; glycosylation affects interaction dynamics	Frequently upregulated in tumors; influences immune infiltration, metastasis, and therapy response	Targeted indirectly through modulation of adhesion/inflammation pathways	Controls TAM recruitment and spatial organization within the TME	(115, 177–180)
CD83	Ig-superfamily immunomodulatory receptor (non-canonical lectin-associated)	Sialic-acid binding has been reported, although its broader ligand repertoire and immunomodulatory interactions remain incompletely defined.	Mature DCs, activated lymphocytes, monocytes/macrophages	No ITIM/ITAM	Regulates DC maturation, antigen presentation, and T cell priming; soluble CD83 promotes tolerogenic signaling	Associated with immune regulation and tolerogenic DC states in cancer	Investigated in immunomodulation contexts; emerging relevance in cancer	May influence TAM/DC crosstalk and tolerogenic programming	(115, 181–185)

The table summarizes receptor classification, ligand specificity, expression, signaling, functional output, cancer relevance, therapeutic modulation, and relevance to TAMs. The I-type lectin family is represented principally by Siglecs; related receptors are included where relevant to myeloid regulation. Ligand recognition and functional consequences may vary with carrier context, density, multivalency, and spatial presentation.

## 6 Discussion

### 6.1 Conceptual limitations in studying glyco-immune regulation

Despite growing recognition of glycosylation as a regulatory layer of tumor immunity, the study of glyco-immune regulation has historically developed through isolated receptor–ligand discoveries rather than comprehensive mapping of lectin-mediated immune programs (2, 137). Many axes were characterized because specific receptors or ligands emerged as relevant in specific cancer contexts, with their lectin biology subsequently connected to immune regulation (94, 95, 105). This has produced a field rich in mechanistic examples (e.g., CD24–Siglec-10, MUC1–Siglec-9, Tn/STn–MGL) but still limited in its capacity to uncover how glycan-lectin interactions operate as coordinated systems shaping immune states (2, 70, 94, 95).

This limitation is particularly relevant in myeloid biology, where cellular phenotypes rarely emerge from a single pathway (2, 3). Tumor-associated macrophages integrate cytokine signals, metabolic constraints, stromal cues, and receptor-ligand interactions into coordinated functional states (2, 3). Glycan–lectin signaling is embedded within this broader regulatory network (3, 137). A single myeloid cell may express multiple lectins, while tumor and stromal compartments simultaneously display multiple glycan ligands (2, 85, 86). As a result, the functional outcome of lectin engagement is unlikely to reflect one receptor in isolation, but rather the combined effect of co-expressed receptors, ligand availability, signaling thresholds, and tissue context (2, 137).

This creates a conceptual gap between current knowledge and biological reality. Glyco-immune checkpoints are often described as discrete inhibitory axes, but their operation in the TME is better understood as combinatorial (2, 85, 86). Multiple lectins can converge on shared outputs, including reduced phagocytosis, impaired antigen presentation, altered cytokine production, metabolic adaptation, and reinforcement of tolerogenic myeloid programs (3, 43, 86). In parallel, the functional output of a given lectin is context-dependent, shaped by ligand density and multivalency, cellular differentiation state, and the surrounding cytokine, metabolic, and spatial environment (4, 90). Receptor-centric studies remain essential for defining binding specificity and signaling mechanisms, but whether individual lectin axes are sufficient to determine myeloid cell states within the tumor microenvironment remains unsolved. Such approaches often fail to capture the cellular organization, carrier context, glycan density and multivalent presentation required for physiologically relevant lectin engagement, and downstream immune programming (2, 3, 81, 137).

A second limitation concerns redundancy. Many immunosuppressive pathways in the TME are organized as partially overlapping networks rather than linear cascades (43, 137). Blocking a single receptor or ligand may therefore be insufficient when suppressive myeloid states are maintained by redundant signaling pathways, compensatory lectin–ligand interactions, and phenotypic plasticity (2, 43, 86). In this context, alternative receptors, overlapping ligand specificities, and parallel cytokine or metabolic cues may preserve the same functional outcome despite disruption of an individual axis (2, 3, 137). The unresolved question is therefore not simply which lectins are present, but how lectin co-expression, ligand diversity, and compensatory signaling combine to stabilize immunosuppressive myeloid programs (2, 3, 86).

### 6.2 Experimental barriers to uncover glyco-immune regulation

The coordination of transmembrane lectin repertoires across macrophage differentiation, tumor context, and immune state remains incompletely understood. Resolving this problem requires experimental designs capable of preserving the cellular organization, ligand presentation, and multicellular signaling dynamics through which glycan–lectin interactions acquire functional meaning, since glyco-immune regulation is unusually sensitive to experimental context (2, 3, 137). Glycan presentation is not only determined by gene expression, but rather depends on cell state, glycosyltransferase activity, metabolic substrate availability, membrane organization, protein carrier identity, and spatial presentation at the cell surface (2, 137, 138). Lectin engagement is similarly dependent on receptor density, glycan density, multivalency, cell–cell proximity, and the physical organization of interacting cells (2, 81, 137). Thus, the limitations of reductionist experimental systems do not arise from a single missing feature, but from the progressive loss or separation of the spatial organization, cellular complexity, human specificity, molecular resolution, and functional perturbation required to accurately capture both the ligand landscape and the consequences of receptor engagement.

Conventional two-dimensional monocultures remain limited for this purpose. They provide experimental control and are useful for dissecting individual ligand–receptor interactions, but they poorly reproduce the spatial constraints that define glyco-immune signaling in tissues (2, 139). Cells grown on rigid plastic experience non-physiological polarity, altered adhesion, simplified nutrient and oxygen exposure, and distorted cell–cell contact geometry (139, 140). These conditions can modify glycosylation programs and membrane organization, thereby altering the density, accessibility, and multivalent presentation of glycan ligands (2, 81, 137). In immune–tumor assays, this limitation becomes particularly consequential because immune cells encounter tumor cells in an artificially accessible plane, without the physical barriers imposed by three-dimensional tissue architecture, extracellular matrix organization, stromal compartmentalization, or gradients of oxygen and metabolites (139, 140). As a result, 2D systems may overestimate immune accessibility while underrepresenting immune exclusion, localized ligand presentation, and niche-specific macrophage polarization (140, 141).

Three-dimensional co-culture tumor models provide an important advance as they better reproduce tissue-like architecture, oxygen and nutrient gradients, matrix constraints, and heterotypic cell interactions (139, 140, 142). Spheroids, organoids, microencapsulated cultures, extracellular matrix-embedded systems, and microfluidic models can preserve aspects of cell polarity, spatial organization, and multicellular crosstalk that are absent from 2D cultures (140–143). For glyco-immune regulation, these features are not only architectural but mechanistic, as they determine whether lectin-expressing myeloid cells encounter glycan ligands in the correct cellular interface and with sufficient density or multivalency to trigger signaling (2, 81, 137).

However, restoring three-dimensional architecture alone is not sufficient. Glyco-immune regulation has a multicellular origin, and tumor cells are not the only source of relevant glycans or lectin-regulating cues (2, 3). Stromal, immune and endothelial cells, as well as extracellular matrix components contribute to the landscape of the TME, shaping both glycan availability and lectin engagement (140). Accordingly, many spheroid or organoid platforms remain limited if they are tumor-centric, lack stromal complexity, or only introduce immune cells as short-term effectors into pre-formed tumor structures (143, 144). Such approaches can model infiltration or cytotoxicity but may not recapitulate the progressive differentiation of monocytes into TAM-like states under continuous exposure to tumor, stromal, metabolic, and matrix-derived cues (141, 144). This distinction is crucial as lectin expression is dynamic and linked to macrophage polarization (86). Models composed of only one or two cellular compartments, or short-term immune–tumor assays, may therefore identify direct mechanisms but miss the network-level interactions through which spatial organization and multicellular crosstalk condition long-term myeloid remodeling and stabilize suppressive lectin programs (140, 141, 144).

Animal models introduce a different set of limitations. They retain systemic immunity, vascularization, stromal remodeling, and tissue-level complexity, but species-specific differences in glycosylation machinery, and lectin repertoires complicate translation (143, 145, 146). This is particularly evident for Siglecs, where CD33-related family members have evolved rapidly and differ substantially between humans and mice (92, 146). Differences in sialic acid biology, receptor orthology, expression patterns, and ligand specificity mean that murine models cannot fully reproduce human lectin-mediated immune regulation (90, 146). As a result, mechanisms inferred from mouse tumors may not map directly onto human myeloid glyco-immune checkpoints.

Models incorporating human tumor or immune components such as humanized mouse models and patient-derived xenografts partially address these limitations but they introduce hybrid-system challenges (145). Human immune reconstitution is often incomplete, myeloid development may be altered, and cross-species interactions between human immune cells and murine stroma can distort cytokine signaling, glycosylation, and tissue remodeling (143, 145). While these models are valuable for assessing systemic immune behavior, they still pose a constraint for dissecting human-specific glycan–lectin interactions in a controlled and mechanistically interpretable way (90, 145).

Patient-derived organoids and ex vivo tumor explants move further toward human tumor fidelity by preserving patient-specific architecture, molecular heterogeneity, and, in some cases, native glycosylation patterns (143, 147). Despite increased biological fidelity, organoids frequently lose immune and stromal compartments during expansion, whereas explants preserve native tissue organization more directly but are short-lived, technically demanding, and difficult to perturb systematically (143, 147). Both systems are constrained by tissue availability, inter-patient variability, low throughput, and increased handling complexity, limiting their suitability for broad comparative analyses or controlled mechanistic dissection (143, 147). For glyco-immune regulation, where causal interpretation requires both patient-level heterogeneity and functional perturbation, these constraints create a need for complementary approaches capable of defining causal regulatory axes (2).

At the hypothesis-generating level, public bulk transcriptomic cohorts and single-cell datasets offer complementary strengths: bulk RNA-seq provides sequencing depth and population-scale coverage, whereas single-cell RNA-seq transformed the ability to resolve lectin expression within defined immune and stromal compartments (148, 149). Together, these approaches can identify recurrent associations between lectin repertoires, myeloid states, and tumor phenotypes, thereby prioritizing candidate axes for experimental interrogation (2, 148, 149). However, transcriptomic data remain insufficient for glyco-immunology. They can infer lectin expression, myeloid cell states, and, to some extent, glycosylation potential through the expression of glycosyltransferases, glycosidases, and other biosynthetic enzymes (2, 138). However, they cannot determine the final glycan structures generated, their linkage and branching patterns, the carrier proteins or lipids on which they are displayed, receptor occupancy, ligand multivalency, or protein-level receptor availability (2, 138). Many glycosylation changes arise from enzyme activity, substrate flux, Golgi organization, metabolic state, and post-translational processing rather than transcript abundance alone (138). RNA-based analyses are therefore essential for identifying candidate receptors and regulatory programs, but they remain insufficient to establish functional glycan–lectin engagement without protein-level validation and experimental perturbation (2, 148).

Spatial technologies address part of this problem by preserving tissue organization and allowing receptor- and ligand-associated compartments to be mapped in relation to each other (150–152). Yet spatial transcriptomics or imaging alone cannot fully establish causality (152). Co-localization does not prove receptor engagement, and expression does not prove functional signaling (150). Moreover, validation of the ligand side of glyco-immune interactions remains technically challenging. Lectin receptors can often be assessed using antibodies by flow cytometry, immunofluorescence, or immunohistochemistry, but equivalent reagents for defined glycan structures are more limited (2, 153). Glycan-directed antibodies are not available for many epitopes, may lack sufficient validation, or may recognize related structures with overlapping motifs (2, 153). Lectin-based probes and glycan-binding reagents can profile glycan classes, but their specificity often reflects motif recognition rather than unique structural assignment and is influenced by glycan density, accessibility, and presentation (81, 153, 154). The challenge is thus not the absence of technologies, but the lack of integrated experimental designs that combine cellular resolution, spatial context, molecular depth, glycan-specific validation, and functional perturbation (148, 151).

Complementary glycomic technologies are therefore required to interrogate the ligand side of glyco-immune regulation (138, 153). Glycan microarrays define lectin-binding specificity and help map receptor–glycan preferences, while lectin-based arrays provide higher-throughput profiling of glycan motifs across biological samples (153, 154). Mass spectrometry-based glycomics and glycoproteomics add structural resolution by identifying glycan compositions, linkage features, and glycosylated carrier proteins, whereas emerging spatial glycomics approaches preserve tissue localization and reveal how glycan motifs are distributed across tumor, stromal, and immune niches (138, 155, 156). However, these technologies also have limitations: they often require specialized workflows, may lose cellular resolution during sample processing, and do not by themselves establish receptor engagement or immune function (155, 156). Thus, glycomics provides an essential ligand-focused layer, but causal interpretation still requires integration with transcriptomic mapping, spatial context, protein-level validation, and functional perturbation (138, 148).

Thus, the central limitation is not the absence of models or technologies, but their fragmentation across biological layers. Tumor glyco-immune regulation emerges at the interface between glycan structure, spatial presentation, lectin expression, myeloid differentiation, and functional immune output, with no single system capturing all these dimensions simultaneously (2). Progress therefore depends on integrated experimental designs that connect discovery-scale profiling with human-relevant multicellular models, protein-level validation, and functional perturbation.

### 6.3 Unresolved knowledge gaps

Together, limitations in both current conceptual frameworks and experimental systems leave several key questions unresolved in glyco-immune regulation. First, there is no systematic map of transmembrane lectin expression across tumor-associated myeloid states in human-relevant cancer models (3, 86). Individual receptors such as MGL, DC-SIGN, Siglec-9, Siglec-10, or CD206 have been studied in specific contexts, but their coordinated expression across macrophage differentiation trajectories remains poorly defined (86, 94, 95, 127).

Second, the field has limited understanding of lectin co-expression and redundancy (2, 137). It remains unclear whether suppressive macrophage states are driven by a dominant lectin axis or by distributed receptor programs in which several lectins compensate for each other (86, 137). This distinction has direct therapeutic implications, as suppressive states may be maintained by redundant lectin pathways, coordinated receptor combinations, or both, limiting the durability of single-receptor targeting and supporting the need for multi-axis or upstream intervention strategies (2, 43).

Third, the integration between lectin repertoires and canonical TAM programs remains incomplete (3, 44). TAM states are often characterized using cytokine profiles, scavenger receptors, antigen presentation markers, angiogenic mediators, metabolic programs, or tissue-remodeling signatures, while lectins are rarely incorporated systematically into these frameworks (3, 44). As a result, it remains unclear which lectins act as functional drivers of macrophage polarization, which reflect established differentiation states, and how these receptors integrate with broader TAM programs (44, 86).

Fourth, functional validation remains limited in human-relevant systems (139, 141). Many glyco-immune mechanisms have been identified through receptor blockade, ligand presentation, or mouse tumor models, but fewer studies integrate population-scale data with perturbation of human multicellular models and protein-level validation (2, 3, 139). This limits the ability to distinguish correlative receptor expression from functional involvement in macrophage polarization, tumor interaction, or immune suppression. Recent work in glioblastoma illustrates this gap: network-based analyses identified ASGR2 and CLEC12A as prominent CLR hubs associated with immunosuppressive myeloid populations and combined poorer prognosis, with protein expression subsequently validated in patient-derived myeloid subsets (124). However, their causal contribution to myeloid immunosuppression remains unresolved.

Finally, there is no clear framework for identifying druggable multi-axis targets in glyco-immune regulation (2, 43). Current therapeutic approaches often focus on individual checkpoints or isolated glycan–lectin pairs, including receptor-blocking antibodies, glycomimetic antagonists, or tumor desialylation strategies (43, 113). However, if suppressive myeloid states are stabilized by coordinated receptor programs, then effective intervention may require targets capable of modulating several outputs simultaneously, including phagocytosis, antigen presentation, cytokine production, and tissue remodeling (2, 43).

Together, these gaps indicate that the central challenge is not simply to identify additional lectin receptors, but to resolve how lectin repertoires are organized, regulated, and functionally integrated within human tumor-associated myeloid states in ways that can be mechanistically tested and therapeutically exploited.

### 6.4 Future directions for mechanistic and therapeutic translation

Addressing the identified knowledge gaps requires a shift from isolated receptor–ligand characterization toward integrated analysis of lectin repertoires, glycan landscapes, and myeloid cell state (2, 137). Population-level datasets can identify recurrent lectin-associated patterns across tumors, experimental systems, or patient cohorts with biological relevance, while single-cell and spatial approaches can assign these patterns to defined immune populations, macrophage states, and tissue compartments (148, 151). These discovery layers are essential for moving beyond individual examples, but they do not by themselves establish functional glycan–lectin engagement. Candidate lectin programs must therefore be connected to receptor abundance, membrane localization, ligand accessibility, and downstream immune function (2, 138).

A human-relevant multicellular context is central to this effort (140). Glyco-immune regulation depends on sustained communication between tumor, stromal, and myeloid compartments, as well as on the spatial constraints that determine whether glycans and lectins encounter each other in biologically meaningful interfaces (3, 140). Models used to study these pathways should therefore preserve tumor–stroma–myeloid crosstalk, support macrophage differentiation under sustained microenvironmental cues, and allow functional perturbation of candidate receptors or regulatory pathways, quantitative phenotyping, and validation of receptor expression at the protein level. Without these features, lectin expression has limited biological significance, as it cannot establish whether a receptor actively contributes to suppressive myeloid programming or merely reflects macrophage differentiation state.

A complete framework must also incorporate the ligand side of the axis (138, 153). Transcriptomic data can suggest altered glycosylation potential through changes in glycosyltransferases, glycosidases, or glycan-processing pathways, but they cannot define the exact glycan repertoire displayed at the cell surface (75, 138, 153). Glycomic and glycoproteomic approaches are therefore necessary to characterize the glycan motifs, linkages, and carrier proteins available for lectin engagement (138, 153, 156). Integrating lectin expression with glycan availability would help determine which receptor–ligand interactions are plausible within a given tumor context, and which represent functional regulatory axes (81, 154).

This integrated view is crucial for therapeutic translation (43, 86). Since suppressive myeloid states are maintained through coordinated lectin pathways, glycosylation patterns, signaling nodes, and macrophage differentiation states, therapeutic strategies focused on single lectin-glycan pairs are unlikely to fully disrupt the underlying immunosuppressive state (2, 43). Durable reprogramming will therefore require approaches that target upstream regulators or multi-axis vulnerabilities capable of modulating several suppressive outputs simultaneously (43). In this context, the goal is not simply to expand the catalog of lectins associated with cancer, but to define which lectin axes are recurrent, ligand-supported, cell-type specific, and functionally validated as drivers of immune suppression.

Thus, addressing glyco-immune dysregulation in cancer requires integrated, multi-layered approaches in human-relevant systems. Transmembrane lectins should be understood not as isolated glycan-binding receptors, but as coordinated sensors and regulators of the tumor microenvironment (2, 137). Defining how their repertoires interact with glycan presentation and macrophage differentiation will be essential for moving from descriptive glyco-immune associations toward actionable mechanisms of myeloid-mediated immunosuppression that guide new therapy development (3, 43, 137).
